# 

**Published:** 1887-09

**Authors:** 


					﻿/olume LV )
SEPTEMBER, 1887,
[Number
^(5y '
^EDIC-AL
' Journal.
EXAMINER
EDITOR:
S. J. JONES, M.D., LL.D
, W'ftARK & LONGLEY (Q,(jmo,l
.....	PVBUSHERS.......k,.t
				

